# Progestogen metabolites for use in pregnancy monitoring of 13-lined ground squirrels (*Ictidomys tridecemlineatus*)

**DOI:** 10.1530/RAF-20-0071

**Published:** 2021-04-08

**Authors:** Amy Miller, Elainna Jentz, Cassandra Duncan, Dana Merriman

**Affiliations:** 1 Department of Biology, University of Wisconsin Oshkosh, Oshkosh, Wisconsin, USA

**Keywords:** sciurids, hibernation, progestogen metabolites, ELISA, pregnancy

## Abstract

13-lined ground squirrels (TLGS; *Ictidomys tridecemlineatus*) are small, omnivorous, fossorial, hibernating sciurids. TLGS are seasonal induced ovulators, with a ~28-day gestation period. The main goal of this study was to ascertain whether enzyme-linked immunosorbent assay (ELISA) of TLGS fecal samples can be used to non-invasively detect pregnancy. Competitive ELISAs for progestogen metabolites were conducted on feces collected from a group of (*n* =13) females. Feces were collected thrice weekly during the breeding season and frozen for subsequent analysis. Competitive ELISAs were run using progesterone kits ), setting data against seven different time-points between hibernation, emergence, and litter birthdate. Eleven females produced litters. ELISA data from the (*n* = 2) non-pregnant females demonstrated no rise in progestogen metabolites at any point over 28 days. In contrast, data from the (*n* = 11) pregnant females all demonstrated a pronounced rise in progestogen metabolites, with most animals displaying progesterone withdrawal in the final week of gestation. A >20-fold rise in progestogen metabolite was observed halfway through gestation (*P* < 005). Analysis on litter size and progestogen metabolite concentration showed no significant correlation (r_2_ = −0.615). Initial correlation analysis done on sex ratio of litters vs progestogen metabolites showed no significant effect of progesterone on sex ratios (males: r_2_ = −0.772, females: r_2_ = 0.375). This work demonstrated that TLGS also undergo progesterone withdrawal about a week before parturition. We have ascertained that a commercially available progesterone assay kit can detect a significant elevation in progestogen metabolites in this species about halfway through gestation.

**Lay summary:**

This research was conducted to discover whether pregnancy prediction is possible in female 13-lined ground squirrels (TLGS; a small hibernating ground squirrel named for their number of stripes). Pregnancy status in this species, we postulated, could be anticipated by generating profiles for individuals via a non-invasive technique known as fecal endocrine hormone profiling. Fecal samples were collected from 13 females thrice weekly for 4 weeks post-hibernation in the breeding season of 2016. Fecal samples were then processed and run through an assay known as an ELISA giving concentrations of hormone metabolites excreted through feces. We then set these samples against time points to develop a profile for each female. We have ascertained that elevated progesterone (potential pregnancy) can be detected by a commercially available assay kit. Understanding hormone patterns in animals gives researchers a better idea of best husbandry practices, including breeding in managed care.

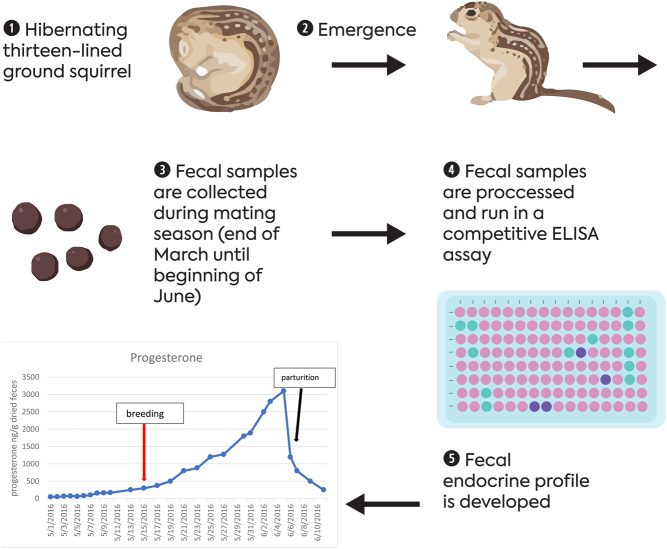

## Introduction

The 13-lined ground squirrel (TLGS; Ictidomys tridecemlineatus) is a rodent species native to most of central North America with a habitat that includes short-grassy areas (such as golf courses, pastures, lawns, and cemeteries) and is an important model species for studies of hibernation biochemistry and physiology (Vaughan *et al.* 2006, Berg von Linde *et al.* 2015, van Breukelen & Martin 2015, Jastroch *et al.* 2016, Ratigan & McKay 2016, Reilly & Franklin 2016, Tupone *et al.* 2016). Hibernation research has led to medical treatments in fields such as organ transplantation, cardiology, and neurology (Arendt *et al.* 2003, Cai *et al.* 2004, Lindell *et al.* 2005). The use of TLGS as a model species has also been central to our understanding of diurnal, cone-dominant vision (Van Hoosier & Nelson 2006, Krubitzer & Stolzenberg 2014, Merriman *et al.* 2016). Investigation into the reproductive physiology of TLGS will help foster new discoveries in these fields.

### Lifestyle and history

TLGS have historically been challenging to breed in captivity (Vaughan *et al.* 2006, Merriman *et al.* 2012), in part because ground squirrel physiology is strongly influenced by circannual rhythms (Helm *et al.* 2013). Therefore, TLGS in the UW Oshkosh animal colony (The Colony) have been bred following replication of the circannual cycle by facilitating hibernation for breeding individuals. TLGS reach sexual maturity at 1 year of age and can produce offspring for at least 6 years thereafter (D. Merriman, unpublished). Additionally, biological cues shared between males and females are thought to promote estrus (Millesi *et al.* 2000), therefore, opposite sex individuals in managed populations are often housed within proximity of each other. In common with wild animals captive male emergence in managed settings is scheduled before female emergence to allow for the resumption of spermatogenesis (Streubel & Fitzgerald 1978). In wild and managed populations males will mate multiple females, and each female produces a single litter of 4–14 young (Johnson 1931). Generally, females produce only one litter a year though a second litter has been reported in the wild in their southernmost range (McCarley 1966), and a second litter has been observed in managed populations within a breeding season only when the first litter perished and the females were immediately re-bred (McCarley 1966).

### Reproductive anatomy, physiology and behavior

The TLGS’s mating strategy is scramble competition polygyny with first male advantage (Foltz & Schwagmeyer 1989). Upon emergence from hibernation in the wild TLGS ovaries already contain antral follicles (Foster 1934), and behavioral estrus lasts a few hours once copulation has occurred (Foster 1934). Females are considered induced ovulators with ovulation occurring 10–36 h post-copulation (Foster 1934). Gestation is estimated to range 27–30 days and variability may be due to differences in ovulatory lag (Johnson 1931, Johnson & Wade 1931, Johnson *et al.* 1933, Foster 1934, Bridgwater 1966, Barr & Musacchia 1968, Landau & Holmes 1988, Vaughan *et al.* 2006, Merriman *et al.* 2012). Copulatory plugs do occur in TLGS but are not often witnessed (Koprowski 1992) and are therefore unreliable as copulation (and thus potential pregnancy) indicators in a large colony.

### Progesterone and parturition/post-parturition in TLGS

Progesterone withdrawal has been demonstrated prior to parturition in several sciurid species (Concannon *et al.* 1984, Holekamp *et al.* 1988, Exner *et al.* 2003), and this is believed to be generalized to all sciurids (Nnamani *et al.* 2013). To permit parturition progesterone signaling is thought to fail in one of two basic ways: either progesterone withdrawal, or functional withdrawal (Mitchell & Taggart 2009).

### Objectives

Regular phlebotomy on an animal with an average 150 g weight is not practical. Handling of animals this small is technically difficult and raises the serum concentration of hormones such as corticosterone, ACTH, corticotropin, and other glucocorticoids which can disrupt reproductive hormone pathways (Reburn & Wynne-Edwards 2000). The use of barbiturates for anesthesia to obtain serum samples have been met with varying success (Reburn & Wynne-Edwards 2000). Therefore, a non-invasive method for determining progesterone patterns pre-breeding and throughout gestation was the main goal of this research, and specific objectives included: (1) validate the use of the Arbor Assays progesterone assay for use in the quantification of progestogen metabolites in the TLGS, and generate reproductive profiles of breeding-season females for use in diagnosing pregnancy and monitoring reproductive status, (2) to determine if TLGS exhibit a progesterone withdrawal that is akin to other sciurids and (3) uncover useful information that progesterone patterns might reveal such as correlations between progestogen metabolites concentrations and litter size, as well as sex ratio of offspring.

## Methods

### Animals and housing

All animal procedures were preapproved by the University of Wisconsin Oshkosh Institutional Animal Care and Use Committee (Approval numbers 0026-000260 and 0026-000288) and conformed to USD A, OLAW, and AAALAC guidelines for rodent care.

The Animal Colony at UW Oshkosh is a large-scale breeding operation used to fulfill the needs of researchers for TLGS. The Colony had ~160 individuals (105 females and 55 males) in the breeding season of 2016.

TLGS were caged in transparent plastic shoebox type cages measuring 43 × 61 × 20 cm and provided *ad libitum* water, a base diet of dog kibble (4–5 pieces daily; IAMS Chunks, Mason, OH, USA) and daily rotating enrichment treats (live mealworms, sunflower seeds, dried vegetables, peanuts in the shell, dried corn) (Van Hoosier & Nelson 2006, Krubitzer & Stolzenberg 2014).

Animals were maintained at the same temperature (22–24°C) and a photoperiod (Beginning April 10, 2016: sunrise 06:16:47 h and sunset 19:33:25 h. Ending May 4,2016: sunrise 05:39:22 h and sunset 20:02:17 h) that mimicked seasonal conditions and fluctuations in natural habitat. Animals were uniquely identified by microchips.

Female TLGS (*n* = 13) were proven dams having weaned litters the year prior. One individual was removed from the study due to illness. Of the remaining females seven were wild-caught and at least 3 years old, and six were captive-bred and 2–3 years of age. Females were removed from the hibernaculum and single-housed after a hibernation period of 28–32 weeks. All 13 aroused from hibernation normally (Wade 1930) and resumed eating the same day. An initial fecal sample was collected the same day as arousal (day 0) from each female while she was single housed. Thereafter, fecal sample collection occurred approximately three times per week until a litter was observed or until 1 June (this was the last date any of the study females could have had a litter based on when the females were separated from the males). All males (*n* = 6, all captive born) were proven sires, emerged from hibernation in a normal fashion, and had enlarged breeding-season testes.

After initial fecal collection, females were exposed to a male within a trio (1M2F) or a quad (1M2F2F) cage. Animals were housed in this fashion because there were fewer males than females in the Colony. In the latter, on alternate days the male was moved back and forth between two pairs of co-housed females. Females were bred to the same male as the prior season whenever possible (6 of the 13 females). While some females rejected the approach of a mate upon introduction inter-individual aggression was rare. Following male exposure of 3.5 weeks females were moved to individual housing (~3 days short of the earliest possible litter birthdate).

### Sample collection and storage

Fecal sample collection commenced at 08:00 h. Squirrels were handled in the same manner; person and order every time. Collection consisted of the female being placed in a transparent plastic shoebox cage measuring 25 × 48 × 20 cm containing a plastic hiding tube and water bottle but no bedding. An enrichment treat (shelled peanut) was offered for occupation and encouragement of GI motility. Females were left in collection cages for 2 h and then all fecal samples were collected. If there were no feces within 2 h that date’s sample was deemed not available (N/A). Fecal samples were placed into Eppendorf tubes and stored at −80°C until extraction, within 4 months from collection.

### Sample selection, extraction, and reconstitution

For the 11 females that gave birth fecal samples were assayed at landmarks with reference to estimated copulation and birthdate. The reason that all samples were not assayed was due to time and resource constraints. These landmarks were: date of pairing but prior to male exposure (DOP), DOP plus 2 days (DOP+2), estimated day of fertilization, that is, 28 days prior to litter birth date (eDOF), litter birth-date minus 21 days (DOB-21), litter birth-date minus 14 days (DOB-14), litter birth-date minus 7 days (DOB-7), and 1-3 days before birth (dependent upon last collection prior to birth to avoid disruption of maternal care).

To determine the overall effect of pregnancy on fecal progestogen metabolites data obtained from the two non-pregnant females during the 4 weeks following male exposure were averaged, and data obtained over the 4-week pregnancies of ten pregnant females were averaged (the 11th parous female was omitted because we did not obtain fecal samples at every landmark time point from her).

Fecal samples were laid onto a square of aluminum foil and placed into a drying oven at 75°C for 2–3 h. Dried samples were immediately ground into a powder with a mortar and pestle. Macro-impurities were removed with forceps and powdered samples were weighed to the nearest 0.01 g. Samples were extracted as per progesterone kit instructions (Arbor Assays DetectX item K025, Ann Arbor, MI, USA). Briefly, 1 mL of 90% ACS grade ethanol was added per 0.1g of feces and shaken for 30 min and aliquots of 100 µL were used for further processing.

Excess was stored at −80°C for re-testing when necessary. Tubes containing 100 µL aliquots of sample were uncapped and allowed to evaporate within a SpeedVac (Labconco Centrivap Concentrator & Cold Trap, model #225638, Kansas City, MO, USA with a Precision Scientific Vacuum Pump, model #D 75, Chicago, IL, USA) and then covered and stored in a freezer dessicator (Thermo Fisher Scientific, product #08-615A, Waltham, MA, USA) for up to a week before reconstitution. Anhydrous ACS Ethanol (90%, 100 µL) was added to each sample and samples were vortexed three times for 10−20 s. ELISA was conducted within 1 week of extraction.

### Competitive ELISA procedures

For ELISA progestogen metabolite kits designed for use with fecal extracts were used. Sensitivity was 47.9 pg/mL and the limit of detection was 52.9 pg/mL. Sensitivity was calculated by comparing the optical densities (OD) for 20 wells run for each of the B0 and standard #7. The detection limit was determined at two standard deviations from B0 along the standard curve. See kit instructions for cross-reactivities (Arbor Assays DetectX item K025, Ann Arbor, MI, USA). Plates were read on a BioRad iMark plate reader (Hercules, CA, USA). Raw data were imported into an online analysis package (www.myassay.com for Arbor Assays Progesterone EIA kit). Wells were run in triplicate. If the coefficient of variation among triplicate wells was >15%, the outlier was excluded from the results or the same sample was re-run on a new plate.

The average percent for extraction efficiency for exogenous hormones was 82% (R^2^ = 0.9929). The average percent recovery for exogenous hormones was 75.8% (R^2^ = 0.9929). Average intra- and inter-assay coefficients of variation were 9.3 and 14%, respectively.

Parallelism (y = −5E−09x^3^ + 4E−05x^2^ − 0.096x + 101.85, R^2^ = 0.9995) determined that the samples from TLGS were immunologically similar to assay kit progesterone. Pooled samples (*n* = 5) showed that extracts had binding percentages in range (20–80%) when run with 1:32 to 1:128 dilution factors (on a neat – 1:2048 scale). Samples were run at a 1:40 dilution to start and depending on the HRP binding (above or below the 20−80% range) were adjusted accordingly. During gestation some samples need to be diluted more due to a <20% HRP binding (1:200 to 1:300), and some baseline samples that had HRP binding values >80% needed to be diluted less (down to 1:5). Dilution adjustments were made on a case-by-case basis after the initial run obtaining percent bindings.

### Statistical analysis

All statistical analyses were performed in R (R Core Team 2016 Vienna, Austria), and Excel version 1609 7369.2120 (Microsoft Corporation, Redmond, WA, USA). An ANOVA with repeated measures was run to compare progestogen metabolites at landmark time points for each female. A pairwise t-test with a Bonferroni correction (α = 0.01) was run to determine where the differences lie.

To determine the effect of litter size on progesterone during gestation, a Pearson’s correlation test was run on seven females and four litter sizes (only these seven had both a full fecal sample set and a confirmed litter size).

A Pearson’s correlation test was run on recorded sex ratios from six of the 2016 litters, vs their respective dam’s progestogen metabolite concentrations to find any potential relationships.

Finally, to determine if the ELISA for progestogen metabolites is an appropriate test to determine levels above baseline in this species a Student’s t-test was used to identify any significant differences between the 11 females that produced litters and the two females that did not (α = 0.05).

## Results

### General study findings

Of the 13 females in this study, 11 bore litters and two did not (85% fecundity). Litter birth dates ranged approximately 18 days, and conception date was determined by assuming a 28-day gestation and counting back from birthdates. These data are in line with what we have historically observed in the Colony. Despite thrice-weekly examination, none of the 11 parous females was ever observed with a copulatory plug.

### Progestogen metabolite patterns during gestation

**Figure 1:**
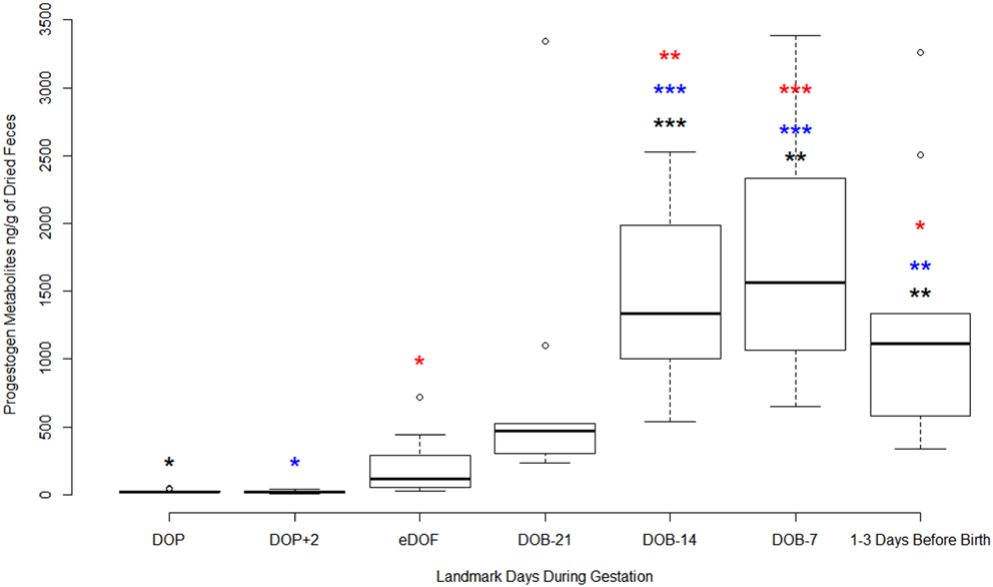
Mean fecal progestogen metabolite data among 10 pregnant 13-TLGS (thirteen-lined ground squirrels; *Ictidomys tridecemlineatus*) females averaged over 4 weeks for all groups. Boxes show the first and third quartile of the data; the dark lines show the median of the data, and the lines above or below the box show the data minima and maxima. Dots that appear above the lines are outliers. Asterisks show significant differences: *0.01, **0.001, ***0.0001, with color coding showing corresponding differences.

Fecal progestogen metabolites of the two females who did not give birth during the study did not rise above baseline (mean for no litter females was 28.85 ng/g of dried feces for the duration of the study). In contrast, fecal progestogen metabolites of the 11 females who did give birth during the study rose substantially above the low initial concentration measured on DOP; averaged results are shown in Fig. 1. The lower mean fecal progestogen metabolite exhibited by non-pregnant females was statistically lower than the higher mean fecal progestogen metabolite exhibited by pregnant females (*P*  = 0.02, *t*_13_ = −3.63). Progestogen metabolite concentrations differed across time points throughout gestation (*P*  < 0.01; Fig. 1). Asterix indicates levels of significant difference. 1, 2, and 3 Asterixis show significant differences between one another. Ex. * is different from ** and ***, and ** is different from ***. Color coding shows corresponding significant differences (Fig. 1). From among individual results the progestogen metabolite concentration measured by competitive ELISA was 4.98 ng/g at DOP+2 days and the highest was 3340.4 ng/g at DOB-21.

A Pearson’s correlation test revealed there was no correlation (rs = −0.615, *P*  = 0.078) between litter size and progesterone concentration.

Pearson’s correlation test showed there was no correlation between number of males in a litter and progesterone (rs = −0.772, *P*  = 0.0717), and no correlation between number of females in a litter and progesterone (rs = 0.464, *P*  = 0.375).

## Discussion

The progestogen metabolite assay kit was validated for the use of detecting an elevation in fecal progestogen metabolite in the TLGS by finding that there was a significant difference between mean concentrations of the pregnant vs non-pregnant females. Profiles of the pregnant females were generated to show the significance of the rise in progestogen metabolite concentration.

The mean fecal progestogen metabolite profile from 11 pregnant TLGS over the breeding and gestation period (Fig. 1) demonstrated steadily rising fecal hormone starting on the estimated day of fertilization (eDOF), peaking approximately 1 week before the litter’s birth (DOB-7), and then falling to about half-maximal levels in the 1–3 days before birth. Compared to the 4 weeks of gestation by pregnant females, 4 weeks of data from non-pregnant females exhibited consistently and significantly lower fecal progestogen metabolite concentration. Therefore, quantifying progestogen metabolites via ELISA proves an effective method for detecting the rise in progestogen metabolites in TLGS stemming from copulation and ovulation, lending toward the possibility of pregnancy.

Diagnosing pregnancy, however, will require more nuanced research since the rise in progestogen metabolites is synonymous with copulation and ovulation, and not necessarily pregnancy. In TLGS the placenta alone is not able to secrete enough progesterone to sustain a pregnancy, and corpora lutea (CL) provide the remainder of the progesterone for pregnancy maintenance. Furthermore, the CL of TLGS remain secretory until at least 2 months post-partum making them a major producer of progesterone, and this study was not able to differentiate between CL progesterone, and placental progesterone (Dripps 1919, Foster 1934). It is worth noting, however, that in this study there were no females that had an elevation in progestogen metabolites that did not result in a pregnancy.

It was found that there is no significant effect of litter size on quantified progestogen metabolites, but further investigation could be warranted with a larger sample size of animals and more frequent serial fecal sample collection.

The same could be said for the correlation tests done on progesterone and sex ratio of offspring; while there were some moderate correlations, neither was significant. However, considering the sample size (*n* = 6) of this small data set may prove important. Collecting and testing more fecal samples from more females may help to tease out these issues.

In Richardson’s ground squirrel (Urocitellus richardsonii) it was found that there was a positive correlation between cortisol and testosterone, and that higher cortisol levels were correlated to smaller litter sizes. Furthermore, they found that the smaller litter sizes had a higher proportion of males (Ryan *et al.* 2014). As far as we could tell there haven’t been any studies to date on litter size effecting progesterone concentration or correlations between progesterone and sex ratio of offspring in ground squirrels let alone TLGS. This, and our study data, point to more work needing to be done on maternal gestational hormone levels and litter data in TLGS.

### Progesterone withdrawal in TLGS

The mean TLGS profile demonstrates classic progesterone withdrawal and agrees well with a study of another hibernating sciurid bred in captivity the Vancouver Island marmot (Keeley *et al.* 2012). The mean of the time point 1–3 days before parturition is significantly lower than the mean of the DOB-7 time point.

### Potential research directions

Due to microbial degradation, fecal steroids can drop by 17% after 2 h at room temperature (Brown *et al.* 2004, Keeley *et al.* 2012) so time lag may have introduced some sample variability during the collection of samples. This means that the signal strength may have been diminished in some samples that may have not been frozen immediately. Future work in this area should require that fecal samples are frozen as soon as possible to lower the risk of signal reduction.

Despite this, the data did indicate how soon after mating we can reliably use elevated fecal progestogen data to distinguish between animals that have copulated and ovulated (and are therefore potentially pregnant) and those who have not copulated and did not ovulate. Future research might employ daily (or even twice daily) fecal collection to determine if a non-pregnant luteal phase could be detected in this species by the same ELISA kit. Currently, there is nothing in the literature that indicates how long a non-pregnant luteal phase is in TLGS.

An iterative process could be performed on future profiles to determine basal and elevated hormone metabolite concentrations (Herrick *et al.* 2010), but was not be performed in this study because of how few samples were analyzed. In all 11 pregnant animals, the mean fecal progestogen metabolites was five-to-ten-fold elevated at eDOF over DOP (Fig. 1). Even more dramatically it was >20-fold elevated at DOB-21 over DOP (Fig. 1). The individual variation recorded at eDOF makes DOB-21 the more conservative choice.

The current study did not include any fecal hormone data on estrogens. Time, money, and man-power restraints did not allow for it but these data indicate the need for more information on estrus and ovulation timing as well as how behavior and hormones correlate. Breeding in managed care would benefit greatly from this information.

## Conclusions

Based on the average fecal progestogen metabolite profile that was generated this study demonstrated that TLGS show a rise in progesterone during pregnancy that can be detected by a commercially available progestogen metabolite ELISA kit. This research also demonstrated that TLGS exhibit a progesterone withdrawal about a week before parturition which is line with other hibernating sciurids. There was no evidence that litter size had any effect on progesterone levels, but more work with a larger sample size is needed. Correlations between sex ratio and progestogen metabolite concentrations showed no correlations between offspring sex ratios and progesterone in TLGS, but more work is warranted here with a larger sample size as well.

## Declaration of interest

The authors declare that there is no conflict of interest that could be perceived as prejudicing the impartiality of the research reported.

## Author contribution statement

Amy Miller: Conceptualization, methodology, software, validation, formal analysis, investigation, writing – original and review/editing, visualization, supervision, project administration. Elainna Jentz: Investigation. Cassandra Duncan: Investigation. Dana Merriman: Conceptualization, methodology, formal analysis, Resources, writing – original/review editing, visualization, supervision, funding acquisition.
